# Development of a New Sequential Extraction Procedure of Nickel Species on Workplace Airborne Particulate Matter: Assessing the Occupational Exposure to Carcinogenic Metal Species

**DOI:** 10.1155/2018/3812795

**Published:** 2018-12-02

**Authors:** Catalani Simona, Fostinelli Jacopo, Gilberti Maria Enrica, Orlandi Francesca, Magarini Riccardo, Paganelli Matteo, Madeo Egidio, De Palma Giuseppe

**Affiliations:** ^1^Unit of Occupational Health and Industrial Hygiene, Department of Medical and Surgical Specialties, Radiological Sciences and Public Health, University of Brescia, Italy; ^2^PerkinElmer (Italia), Milano, Italy

## Abstract

Nickel (Ni) compounds and metallic Ni have many industrial and commercial applications, including their use in the manufacturing of stainless steel. Due to the specific toxicological properties of the different Ni species, there is a growing interest about the availability of analytical methods that allow specific risk assessment, particularly related to exposure to the Ni species classified as carcinogenic. In this paper, we described a speciation method of inorganic Ni compounds in airborne particulate matter, based on selective sequential extractions. The analytical method described in this paper allows the determination of soluble, sulfidic, metallic, and oxide Ni by a simple sequential extraction procedure and determination by Atomic Absorption Spectroscopy using small volumes of solutions and without long evaporation phases. The method has been initially set up on standard laboratory mixtures of known concentrations of different Ni salts. Then it has then been tested on airborne particulate matter (powder and filters) collected in different workstations of a large stainless steel production facility. The method has occurred effectively in the comparison of the obtained results with occupational exposure limit values set by the main international scientific and regulatory agencies for occupational safety and health, in order to prevent both toxic and carcinogenic effects in humans.

## 1. Introduction

Nickel (Ni) compounds and metallic Ni have many industrial and commercial applications, including their use in stainless steel production, in a large series of metal alloys, as catalysts, in batteries, pigments, and ceramics [1]. An industrial sector in which Ni exposure can be particularly relevant is the production of special stainless steel in secondary steel foundries: the workers engaged in this industry are potentially exposed to various forms of airborne Ni, in particular during the operations of melting and casting and at all the stages of the process characterized by the need for high temperatures [2, 3]. This kind of production has been widespread for decades in northern Italy, involving thousands of workers and consequently arousing high interest on the related occupational and public health issues. The toxicological properties of Ni compounds yet represent an important challenge in terms of risk assessment and are also of great concern for the necessary enforcement of preventive measures in exposed workers [4, 5].

Exposure to Ni oxides and sulfides, which have low solubility in water, has been recognized as one of the prominent causes for occupational Ni-related lung and nasal cancer [6, 7].

The carcinogenic potential of water-soluble Ni compounds and Ni tetracarbonyl has been continuously discussed for decades [8]. Although there is no evidence that exposure to metallic Ni increases the risk of respiratory cancer, it is well known as the most important sensitizer among metal elements [9–12].

In 1990, the International Agency for Research on Cancer (IARC) concluded that there were sufficient evidences in humans for the carcinogenicity of Ni sulfate and of combinations of Ni sulfide and oxides in the Ni refining industry [13]. In 2012, with specific referral to the inhalator exposure route, IARC updated the evaluation classifying Ni compounds as “carcinogenic to humans-group 1”, whereas metallic Ni and Ni alloys were categorized as “possibly carcinogenic to humans-group 2B”, specifically for cancer of the lung and the nasal cavity [14].

The current understanding of the carcinogenic potential of the most prominent Ni species in sulfidic Ni (Ni subsulphide (Ni_3_S_2_), Ni oxide (NiO), Ni metal (Ni^0^), and soluble Ni (primarily Ni sulfate, NiSO_4_) has been determined through studies based on a combination of animal testing (of pure compounds) and human epidemiological data [15].

Due to the above-mentioned reasons, the speciation of Ni in workplaces' airborne particulate is of the utmost importance for the assessment of the respiratory health risks.

Regarding occupational exposure limits, different threshold levels for Ni and Ni compounds in workplaces and emissions are available (Table 1). In 1998 the American Conference of Governmental Industrial Hygienists (ACGIH) published separated threshold values for the organic and inorganic forms of Ni [16]. In doing so it was recognized that different Ni species had different toxic and carcinogenic properties [17].

In the “Recommendation from the Scientific Committee on Occupational Exposure Limits” for Ni and inorganic Ni compounds”, by the European SCOEL, some occupational exposure limits (OELs) aiming to protect both from inflammatory effects in the lung and from cancer were published [18].

The determination of the total concentration of Ni, accordingly, gives no information about environmental risks or knowledge of the various forms, which makes monitoring of specific chemical species of Ni in environmental samples, such as airborne particulates, extremely important [19]. Consequently, the development of analytical techniques for the determination of various compounds of metallic elements in environmental samples, such as ambient aerosols, is presently one of the most challenging tasks for environmental analytical chemistry [20, 21].

Ni determination can be performed with various analytical techniques, including spectrophotometry, atomic absorption spectrometry (FAAS and ETAAS), inductively coupled argon plasma optical emission spectrometry (ICP-OES), inductively coupled plasma mass spectrometry (ICP-MS) and voltammetry. In the past two decades many techniques have been widely developed for the speciation of inorganic contaminants in environmental samples [22, 23]. Several of them make use of sequential extraction schemes to determine the metal distribution over different fractions, usually including species such as soluble, sulfidic, metallic, and oxide fractions. The application of sequential extraction procedures provides relevant environmental information.

Several of the sequential extraction procedures found in the literature are variants of the method proposed by Zatka et al. in the early 1990s [24] in which the solubility of the different fractions was utilized for the sequential determination of Ni ions.

The procedure of Zatka involves a sequential leaching of airborne dust from Ni production sites and Ni-using workplaces by using ammonium citrate, hydrogen peroxide/ammonium citrate, and bromine-methanol. Acceptable recoveries were obtained, for most species better than 95%.

Up to now, the sequential extraction procedure proposed by Zatka has mainly been applied for speciation of Ni in work-room air (for instance, in Ni refinery) present at mg/m^3^. These levels however, obtaining correct results for the concentrations of trace elements in out-door air at ng/m^3^ levels, are still a great concern, mainly due to the extremely small amounts of sample and analyte.

## 2. Aims

In consideration of the toxicological properties of Ni compounds, this paper describes a modified method which is mainly based on the fractionation proposed by Zatka et al. [25] but achieving both time optimization and greater sensitivity. The method has been first setup on a mix of different Ni species. Subsequently, the method has been applied on airborne particulate matter sampled in different departments of a steel production facility, in order to assess the airborne levels of different Ni species.

## 3. Materials and Methods

### 3.1. Reagents

All chemicals used were of analytical grade. The water used was bidistilled water, for inorganic trace analysis (Merck KgaA, Darmstadt, Germany).

The reagent in the solution for the elution of different Ni species from the filters was as follows:

(A) Ammonium citrate solution: 1.7% ammonium hydrogen citrate and 0.5% citric acid solution, the solution was prepared by dissolving 1.7g of diammonium hydrogen citrate ((NH_4_)2H-Cit)), CAS No. 3012-65-5, Carlo Erba, Milan, Italy) and 0.5 g of citric acid (99%, C_6_H_8_0_7_, Sigma Aldrich, Saint Louis, Missouri, USA) in 100ml of bidistilled water.

(B) H_2_O_2_ citrate: ammonium citrate 0.1M and hydrogen peroxide 30% (w/w) ratio 2:1 (H_2_O_2,_ CAS No. 7722-84-1 Sigma Aldrich, Saint Louis, Missouri, USA).

(C) Methanol-bromine solution: methanol (CH_3_OH, Chromasolv ≥99.9%, CAS No. 67-56-1, Honeywell, Thermo Fisher Scientific); Bromine (Br_2_, CAS No. 7726-95-6 Sigma Aldrich, Saint Louis, Missouri, USA), 50:1.

(D) Nitric and hydrochloric acid solution: nitric acid (HNO_3_ 70%, CAS No. 7697-37-2 Sigma Aldrich, Saint Louis, Missouri, USA) and hydrochloric acid (HCl 37%, CAS No. 7647-01-0 Sigma Aldrich, Saint Louis, Missouri, USA) ratio 1:1.

The mixture of Ni compounds was prepared by several salts: Ni(II) sulfate hexahydrate (NiSO_4_, PM 262.7, reagent plus® 99.99%, CAS No. 7786-81-4 Sigma Aldrich, Saint Louis, Missouri, USA), Ni sulfide (Ni_3_S_2_, 99.7%, PM 240.1 CAS No. 12035-72-2 Sigma Aldrich, Saint Louis, Missouri, USA), Ni powder (PA 58.7, 100mesh, 99.999%, CAS No. 7440-02-0 Sigma Aldrich, Saint Louis, Missouri, USA), and Ni(II)oxide (NiO, PM 74.7, 99.999%, CAS No. 1313-99-1 Sigma Aldrich, Saint Louis, Missouri, USA).

**Figure 1:**
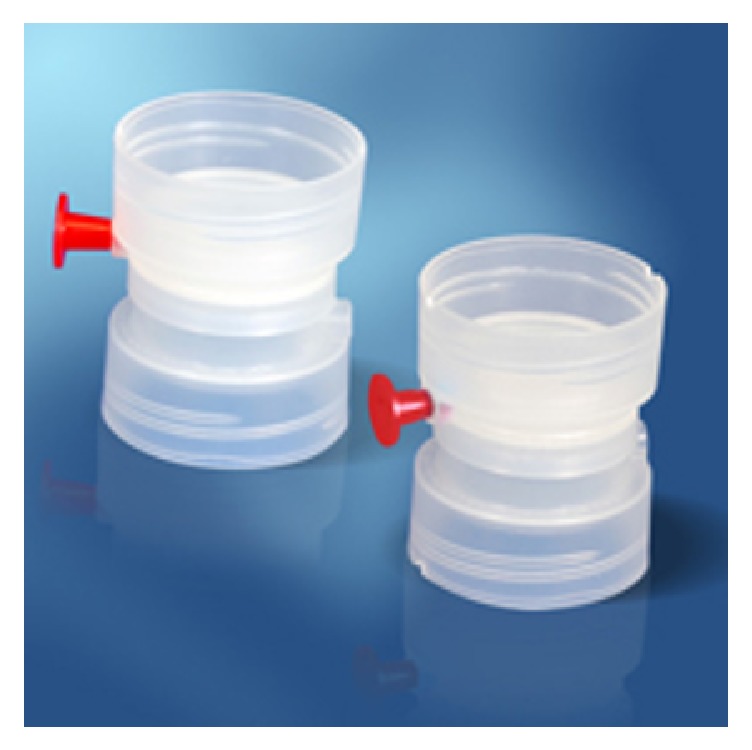
Filter device utilized for sequential extraction of Ni's fractions (DigiFILTER 0.45 micron, SCP Science, Quebec, Canada).

The sequential leaching was carried out in an all hydrophilic Teflon filter holder (DigiFILTER 0.45 micron, SCP Science, Quebec, Canada); the holder was fitted with a 25mm (5*μ*m) PVC filters (SKC Inc. 25mm, 5.0*μ*m) (Figure 1). The filtrates are collected in a test tube (DigiTUBEs 50ml, SCP Science, Quebec, Canada).

A 0.45 micron Teflon membrane inserted in every DigiFILTER guarantees 98% particle retention.

The DigiFILTERs were connected to a vacuum pump; the filtering system is set up so that mild suction can be turned on and off at short intervals if required.

### 3.2. Determination of Nickel

The determination of different fraction of Ni were performed by atomic absorption spectrometry (AAS Spectra 400 Varian, Medical Systems, Inc. Palo Alto, CA) equipped with a transversal Zeeman-effect background correction system and an auto sampler was used for all measurements. The instrumental operating parameters of the AAS apparatus are reported in Tables 2 and 3.

Ni stock standard solutions was prepared from 1 mg/mL (1000ppm) of standard solution (Ni(0) in 2% HNO_3_, O2Si smart solution, Charleston, USA). Working solutions at 0.05; 0.1; and 0.5 mg/L were prepared by serial dilution in bidistilled water of the standard at 1000 mg/L solution (0.05ppm, 0.1ppm, and 0.5ppm).

The accuracy of the method was evaluated by analyzing certified reference materials (NIST 1643e-1643d trace elements in water for ultrafiltrate).

Instrumental limit of detection (LOD) of the total Ni, calculated as three standard deviations of the background signal obtained on 10 blank samples, was equal to 1 *μ*g/L.

The limit of quantification (LOQ) of the total Ni, calculated as ten standard deviations of the background signal obtained on 5 blank samples, was equal to 3 *μ*g/L.

The relative standard deviation (RSDs) of measurements of Ni solutions was between 5 and 10 %.

#### 3.2.1. Extraction Tests

A little amount of all the Ni salts was treated in falcon with one leaching solution at a time and then the concentration of Ni was then determined.

To facilitate the dissolution the falcons were placed in an ultrasonic bath for 5 minutes.

Several tests have been carried out to evaluate different extraction conditions by using different volumes of solutions, times and temperatures (date not shown). The best conditions are reported in Table 4 which illustrates the composition of the solutions, conditions of the extractions, dissolution of the salts, and the recovery of Ni fraction.

The Ni soluble fraction was effectively extracted by all the solutions. The sulfidic fraction was not solubilized by solution A, while it was dissolved by other solutions. The metallic fraction was well extracted by solution C and D. The Ni oxide is solubilized only by a solution HCl:HNO_3_ (1:1); the leaching with the other solutions does not show traces of Ni in solutions.

The same procedure was carried out on all the solutions (A, B, C, D) without the addition of Ni salts.

### 3.3. Speciation Procedure

In order to have Ni concentrations nearer to those found in real samples, each Ni compound was homogenously dispersed and grinded in an agate mortar.

The final mixed salts contained 10.4 mg NiSO_4_ equal to 2.3 mg as Ni, 18.5 mg Ni_3_S_2_ equal to 113.3 mg as Ni, 15.8 mg Ni(0), and 11.3 mg NiO equal to 8.9 mg as Ni.

Quantities ranging from 1 to 2 mg of the mix weighed to the 5th decimal were deposited in a PVC filter placed on the DigiFILTER (Mix A = 0.4 mg; Mix B = 1.3 mg).

**Figure 2:**
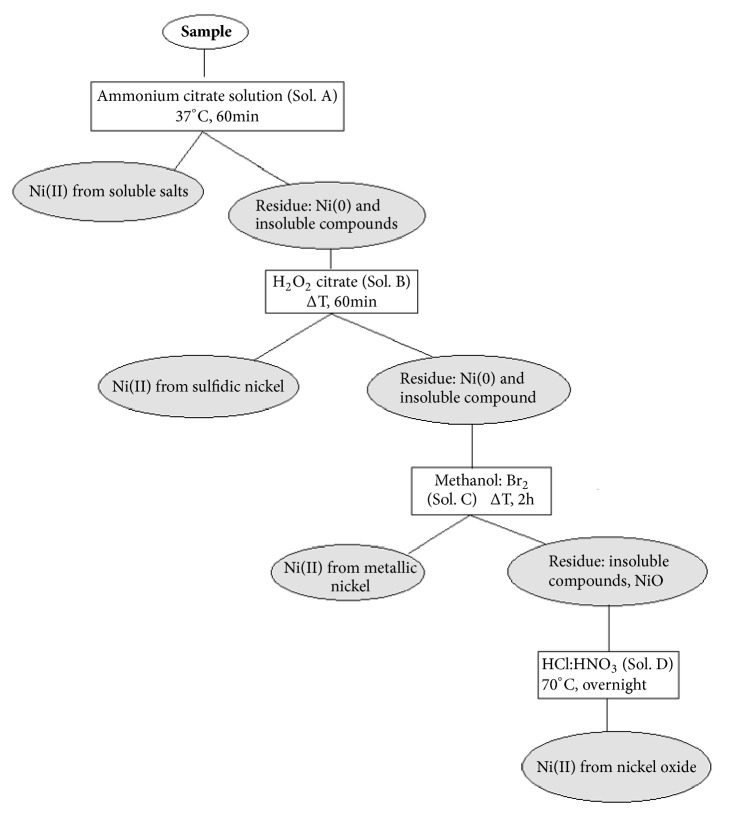
Selective sequential solubilization of inorganic Ni compounds: scheme of the procedure.

The sequential extraction procedures are illustrated in Figure 2 and the results in Table 5.

### 3.4. Determination of Soluble Nickel

Add 10mL of ammonium citrate solution (solution A) in DigiFILTER inserted on a falcon; place the DigiFILTER in the oven at 37°C for 60 minutes. With a vacuum pump, draw the solution into the falcon and keep it for the determination of the soluble Ni fraction. Insert a new falcon in the DigiFILTER.

### 3.5. Determination of Sulfidic Nickel

To the filter from which soluble Ni phases have been leached out add 10mL of the solution H_2_O_2_ citrate: ammonium citrate (solution B). Keep at room temperature for 60 minutes under a hood.

With a vacuum pump, draw the solution into the falcon and keep it for the determination of the sulfidic Ni fraction. Insert a new falcon in the DigiFILTER.

### 3.6. Determination of Metallic Nickel

To the filter from which soluble and sulfidic Ni phases have been leached out add 10mL of the solution methanol:bromine (solution C). Keep at room temperature for 2 hours. With a vacuum pump, draw the solution into the falcon and keep it for the determination of the metallic Ni. Insert a new falcon in the DigiFILTER.

### 3.7. Determination of Oxide Nickel

To the filter from which the first Ni's fraction have been leached, add 5mL of solution HCl:HNO_3_ (solution D) and keep at room temperature overnight under the hood. With a vacuum pump, draw the solution in the new falcon. Transfer the filter from the support into the falcon and add another 5 mL of HCl:HNO_3_ and heat in a water bath at 70°C for 15 minutes.

## 4. Application of Sequential Leaching to Real Samples

### 4.1. Sampling Site and Equipment

The sampling was performed in a steel foundry plant specialized in stainless steels for naval and aerospace industry; the production cycle is based on an electric arc furnace with subsequent casting in a continuous plant. The melting is essentially performed in a three-phase furnace equipped with three graphite electrodes. The different qualities of steel are obtained by mixing recycled scrap with chromium, Ni, and other raw materials. Through the refining in the ladle furnace, specific compositions and quality of the steel are reached (the exact composition of the alloy is proprietary information).

In order to characterize occupational exposure to airborne Ni compounds, we carried out a characterization of Ni species collected on two different types of substrate:

1. Particulate collected through an IOM (Institute of Occupational Medicine) sampler (SKC Inc.) on PVC membrane filters (diameter: 25 mm; porosity 0.5* μ*m), according to Italian standards [26]. The sampling time of the inhalable fraction ranged from three to six hours in each location.
2. Samples of deposition powders generated by industrial processes.

All samples were collected in production areas subjected to possible Ni airborne exposure, i.e., the ladle furnace, the continuous casting area, and the electric arc furnace.

## 5. Leaching and Results

The speciation procedure described in this paper was applied to real samples of environmental dust and filters.

A small amount of collected powder (in the order of a few mg) was placed on a PVC filter and treated with the sequential leaching described above. The results of speciation are reported in Table 6.

The analysis was also carried out on a PVC filter clean to control any forms of contamination.

In all the samples, the most represented species was NiO (35-63%), followed by metallic Ni (7-48%); the soluble and sulfidic fractions were equally distributed (8-30% and 3-23%, respectively). In two samples of powders (A and C) the soluble and sulfidic fractions were not detectable.

In all remove samples, the sum of the different Ni fractions was comparable with the amount of total Ni. The sum of the fraction was slightly lower than total Ni measured in AAS, and the difference was comprised between 1.4 and 19.4%.

At each analysis set, a sample of weighed salts mix is extracted in parallel to verify the quality and effectiveness of the extraction. The quality of determination of total Ni was checked with certified material (NIST 1640, metallic elements in water).

## 6. Discussion

The speciation of Ni in environmental air samples has been debated for several years, since there are threshold limits values relative to the single species but the analytical techniques for determining them are not always available or easy to use. The speciation of Ni can be obtained through chemical leaching and subsequent analysis of the solutes with traditional methods of Ni determinations or by X-ray determination [27]. In the literature several authors have tried to standardize the leaching process and the contrasting results demonstrate the high variability of the method.

The first available method was published by Zatka et al. [28], which, through several washings and dry evaporations, determines the soluble Ni fraction, the sulfidic Ni, the metallic Ni, and the oxide in sequence with a relative standard deviation (RSD) ranged from 3.2 to 3.7%.

Over the years, other authors have tried to replace by using different solutions. Bolt et al. [28] have developed an inline system for extracting soluble Ni compounds (with ammonium citrate buffer), Ni sulfide (with ammonium citrate and hydrogen peroxide), and metal Ni (with CuCl2 / KCl). The final digestion of the residues on the membrane with HNO_3_/HCl leads to the determination of the Ni oxide fraction. The method is based on the one developed by Zatka but requires much less time in execution. In 2003, Profumo et al. [29] reported a method of speciation based on sequential extraction. The authors succeeded in speciating Ni metal and soluble Ni compounds, such as sulfate and chloride and among from the insoluble Ni oxide and sulfide. The Ni recoveries of the different species were in the range 94/99%.

Also Conard et al. [30] tried to improve the sequence of Zatka especially in the separation step of the Ni sulfide/metal phases with leaching with ammonium citrate and hydrogen peroxide increasing the volume of the solution, time, and percolation methods. Despite the studies and tests to date, there is no standardized extraction method to carry out the chemical speciation of Ni, and the proposed methods are difficult to apply on environmental samples.

Our study allowed us to overcome some of the prominent methodological limits of the previously described speciation techniques: the excessive length of the extraction phase and the powder leak during the leaching process.

This method is based on the different solubility based on the different chemical-physical properties of the inorganic Ni species which allow a sequential extraction of the fraction and a determination of Ni.

The test on all the solutions of each single fraction shows how the solutions selectively dissolve the inorganic Ni species.

NiSO_4_, more soluble compound, is dissolved by all four solutions with an efficacy ranging from 89 to 103%, while the less soluble Ni dissolves only in a solution of HNO_3_ and HCl 1:1.

Compared to the previously published methods we reduced the overall volume of the solvents used to only 10 mL, therefore avoiding the evaporation phase and the issues related to the retaining of the solutes. Previous tests on the same sequence of leaching conducted on a filter placed on a simple vacuum extraction device led to very low retaining rates in the order of 50% (date not shown); the adoption of DigiFILTER devices allowed to improve the retaining rate (reaching 94-99%) probably preventing the loss of small particles independently from the different Ni species.

The determination of species of Ni in real powder or filter allowed identifying the different species associated with the different samples.

In all remove samples, the sum of the different Ni fractions was comparable with the amount of total Ni. The sum of the fraction was slightly lower than total Ni measured in AAS; the difference was comprised between 1.4 and 19.4%.

Moreover, taking into account the great progress that has been made in the identification of new health related particle size exposure assessment, the methods described in this paper could also be applied in analyzes carried out on particulate ultrafine material [31]. The value of inhalable Ni aerosols monitoring by species has been widely demonstrated in a variety of working environments as a fundamental tool in assessing respiratory cancer risk [32, 33]. A species-specific approach to setting occupational exposure limits guarantees that the best available health and exposure data will be used. Future research may lead to consideration of setting species-specific OELs on the basis of certain subfractions of inhalable Ni aerosols.

Better Ni speciation techniques will improve the capability of assessing the occupational exposure to carcinogens together with both the measurement of the inhalable fraction and the particle size distribution, as expected and hoped in previous studies [34].

## 7. Conclusion

The analytical method reported in this paper allows the determination of soluble, sulfidic, metallic, and oxide Ni by a simple sequential extraction procedure and analysis by Atomic Absorption Spectroscopy using small volumes of solutions and without long evaporation phases. For the purpose of assessing occupational exposure to carcinogens, the method developed allowed the separation and speciation of the different Ni species on the inhalable fraction of the airborne particulate sampled in different working environments.

The speciation of Ni in real environmental samples allowed us to compare our results with the TLVs proposed by the different agencies and regulations, which is not possible with the sole determination of the total metallic Ni.

## Figures and Tables

**Table 1:** Ni species, with chemical formulas, solubility characteristics, 8h-TWA occupational exposure limits proposed by international agencies, and hazard statements assigned by the EU CLP Regulation, ACGIH, SCOEL, and OSHA.

***Nickel Species***		***Solubility in* ** ***water* ** ***(g/100ml)* ** ***[temperature]***	***CAS Number***	***ACGIH***	***SCOEL***	***OSHA***	***CLP***
**Nickel Sulfate**	NiSO_4_	65.5 [0°C]	7786-81-4	A4 0.1 mg/m^3^ (Ni Sol)	0.01 mg/m^3^ #	-	H351

**Nickel subsulphide**	Ni_3_S_2_	poorly soluble	12035-72-2	A1 0.1 mg/m^3^	0.005 mg/m^3^ § 0.01 mg/m^3^ #	0.1 mg/m^3^ (Ni insol)	H350i

**Nickel monoxide**	NiO	poorly soluble	1313-99-1	A1 0.2 mg/m^3^ (Ni insol)	0.005 mg/m^3^ § 0.01 mg/m^3^ #	0.1 mg/m^3^ (Ni insol)	H350i

**Metallic nickel**	Ni	poorly soluble	7440-02-0	A5 1.5 mg/m^3^	0.005 mg/m^3^ §	0.5 mg/m^3^	H351

#Inhalable fraction; §respirable fraction.

**Table 2:** AAS instrumental parameters for determination of Ni

**Operating conditions**	
Primary source	Nickel Hollow Cathode lamp (Agilent Technologies)

Lamp current	5 mA

Analytical wavelength	232 nm

Background correction system	Zeeman effect based (Transversal)

Slit width	0.2 nm

Mode	Absorbance (peak height)

Graphite furnace operation	

Atomization tube	Partition tubes (coated)-GTA (Agilent Technologies)

Sheath/Purge gas	Argon (Ar) of 99.999% purity

Sample Injection (sample, *μ*L)	30

**Table 3:** Temperature program of the AAS method for determination of nickel.

Step N°	Temperature °C	Time (sec)	Flow (L/min)	Type of gas
1	40	5.0	3.0	Argon

2*∗*	150	35.0	3.0	Argon

3*∗*	150	5.0	3.0	Argon

4^*α*^	900	10.0	3.0	Argon

5^*α*^	900	15.0	3.0	Argon

6^*α*^	900	2.0	0.0	

7^*β*^	2400	1.0	0.0	

8^*β*^	2400	2.0	0.0	

9	2500	1.0	3.0	Argon

10	2500	2.0	3.0	Argon

*∗*Drying step, ^*α*^pyrolysis step, and ^Β^atomizing step.

**Table 4:** Results of dissolution of different nickel fractions (N°=3).

***Nickel Species***	**Solution**	**Treatment**	**Aspect**	**Ni expected** **(mg)**	**Ni determined** **(mg)**	**% extracted**
**NiSO**_**4**_	ammonium citrate (Sol A)	10mL, 37°C, 60min	clear	1.6	1.70±0.1	102%
	H_2_O_2_ citrate: ammonium citrate (Sol B)	10mL, ΔT, 60min	clear	2.3	2.1±0.2	91%
	solution methanol:Bromine (solution C)	10mL, ΔT, 2h	clear	1.8	1.6±0.1	89%
	Solution HCl: HNO_3_ 1:1 (Sol D)	4mL, 70°C, 30min	clear	2.6	2.7±0.3	103%

**Ni**_**3**_ **S**_**2**_	ammonium citrate (Sol A)	10mL, 37°C, 60min	residue	10.8	0.31±0.1	0.03%
	H_2_O_2_ citrate: ammonium citrate (Sol B)	10mL, ΔT, 60min	clear	7.6	7.4±1.1	96%
	solution methanol: Bromine (solution C)	10mL, ΔT, 2h	clear	8.0	8.3±0.8	103%
	Solution HCl: HNO_3_ 1:1 (Sol D)	4mL, 70°C, 30min	clear	7.4	8.8±1.2	118%

**Ni (0) metallic**	ammonium citrate (Sol A)	10mL, 37°C, 60min	residue	4.6	/	/
	H_2_O_2_ citrate: ammonium citrate (Sol B)	10mL, ΔT, 60min	residue	12.2	0.5±0.1	4%
	solution methanol: Bromine (solution C)	10mL, ΔT, 2h	clear	8.7	9.2±1.1	105%
	Solution HCl: HNO_3_ 1:1 (Sol D)	4mL, 70°C, 30min	clear	13.4	15.7±2.3	117%

**NiO**	ammonium citrate (Sol A)	10mL, 37°C, 60min	residue	7.0	/	/
	H_2_O_2_ citrate: ammonium citrate (Sol B)	10mL, ΔT, 60min	residue	13.4	0.1±0.1	1%
	solution methanol: Bromine (solution C)	10mL, ΔT, 2h	residue	7.4	/	/
	Solution HCl: HNO_3_ 1:1 (Sol D)	4mL, 70°C, 30min	clear	10.4	11.8±3.2	114%

**Table 5:** Extraction of specific Ni's compounds in two mixtures of powders. For each Ni species the percentage of recovery with respect to the Ni salt added is reported.

**Ni species**	**Mixture A**		%	**Mixture B**		
	Ni added (mg)	Ni found (mg)		Ni added (mg)	Ni found (mg)	%
**NiSO**_**4**_	0.016	0.013	81	0.054	0.049	91

**Ni**_**3**_ **S**_**2**_	0.096	0.080	83	0.310	0.290	93

**Ni(0)**	0.114	0.11	96	0.367	0.371	101

**NiO**	0.064	0.065	102	0.207	0.220	106

**Table 6:** Determination of Ni's fraction in powders (*μ*g/g) and environmental samples (*μ*g/m^3^) collected in the departments of the steel production facility.

**Powder (** ***μ*** **g/g)**	**NiSO**_**4**_	**Ni**_**3**_ **S**_**2**_	**Ni(0)**	**NiO**	Σ	**Ni Tot AAS**
A (1.2 mg)	/	/	145	196	341	423

B (1.1 mg)	2727	395	745	6673	10540	11509

C (0.1mg)	/	/	323	356	679	695

D (1.0 mg)	580	232	2180	4440	7432	7538

**Filters (** ***μ*** **g/m** ^**3**^ **)**	**NiSO**_**4**_	**Ni**_**3**_ **S**_**2**_	**Ni(0)**	**NiO**	Σ	**Ni Tot AAS**

Filter A	0.32	0.07	0.64	1.5	2.53	2.81

Filter B	0.25	0.67	0.94	1.0	2.86	3.12

Filter C	0.76	0.28	1.6	3.5	6.14	6.44

Filter D	0.87	0.73	4.0	5.0	10.6	10.88

Filter E	0.89	0.24	0.55	1.3	2.98	3.50

## Data Availability

All the data from determinations of Ni species in powders and filters, used to support the findings of this study, are included within the article.
