# Erratum to “Development of a New Sequential Extraction Procedure of Nickel Species on Workplace Airborne Particulate Matter: Assessing the Occupational Exposure to Carcinogenic Metal Species”

**DOI:** 10.1155/2019/6841063

**Published:** 2019-03-03

**Authors:** Simona Catalani, Jacopo Fostinelli, Maria Enrica Gilberti, Francesca Orlandi, Riccardo Magarini, Matteo Paganelli, Egidio Madeo, Giuseppe De Palma

**Affiliations:** ^1^Unit of Occupational Health and Industrial Hygiene, Department of Medical and Surgical Specialties, Radiological Sciences and Public Health, University of Brescia, Italy; ^2^PerkinElmer (Italia), Milano, Italy

In the article titled “Development of a New Sequential Extraction Procedure of Nickel Species on Workplace Airborne Particulate Matter: Assessing the Occupational Exposure to Carcinogenic Metal Species” [1], the first and last names of all the authors were reversed. The revised authors' list is shown above.
